# Logistics hub location for high-speed rail freight transport with road-rail intermodal transport network

**DOI:** 10.1371/journal.pone.0288333

**Published:** 2023-07-11

**Authors:** Siyu Li, Maoxiang Lang, Xinghan Chen, Shiqi Li, Wenqian Liu, Weilin Tang

**Affiliations:** School of Traffic and Transportation, Beijing Jiaotong University, Beijing, China; Libyan Academy, LIBYA

## Abstract

In this paper, we propose a new freight mode to describe how the designed HSR freight train serve for express delivery. We introduce the functions of the hubs and design the hybrid hub-and-spoke network of road-rail intermodal transportation from the perspective of planners, which features as single allocation rule and configures varying levels of hubs. The problem is accurately described by a mixed integer programming model with the objective to minimize total construction cost and total operation cost. We develop a hybrid heuristic algorithm based on a greedy strategy to obtain the levels of hubs, customer allocation and cargo routing. Taking the example of HSR freight network consisting of 50 cities in China, numerical experiments are conducted on the basis of forecasting data from the real-life express market to obtain the hub location schemes. The validity of the model and the performance of the algorithm are verified.

## 1. Introduction

Recent years, the logistics industry has boomed and the volume of express delivery has risen dramatically in China. In order to improve the logistics level and achieve the goal of low carbon, China is vigorously developing the mode of applying high-speed rail (HSR) train to transport express parcels. Parcels arrive at the destination by high-speed rail train and are delivered by road, which is a new mode for express transport called HSR Express. The mode offers higher timeliness and better stability than the road transport.

At present, HSR Express transport has developed organization modes such as parcels transported by dynamic testing trains, parcels carried by one or several cabins of passenger trains and parcels loaded sporadically on the free spaces on passenger trains. However, the freight volume of all these modes is extremely restricted. Dynamic testing trains run regularly, typically once per day. And there are fewer reserved carriages for the other modes. The loading and unloading mode on the passenger platform will inevitably cause interference to passenger transportation. In summary, all three transportation modes have limitations such as inflexible departure times, limited frequency of operation, restricted transport capacity, and lower efficiency. These factors significantly hinder the efficiency of the HSR express. Therefore, there is an urgent need to design a high-speed freight transportation method with a large carrying capacity and minimal impact on passenger transportation. And high-speed trains with all dedicated freight carriages have already been put into production since 2020 [1].

One instance exists where the majority of research findings in the field of high-speed rail express are based on the freight organization at high-speed railway passenger stations, with an emphasis on the theory and application of the operation for the current high-speed rail network. The potential and operation mode of high-speed railway for parcel and mail transportation are discussed by Liang and Wang, Ertem and Keskin, Liang et al., Mathieu, Bi et al. [2–6]. Considering the variation of the demand of China’s express market in the future, the method of "three-stage" forecasting of HSR express cargo flow OD is used by Chen et al. to forecast the demand for HSR express between cities [7]. Yu et al., Yu et al. and Li et al. studied the high-speed rail freight train transport organization under different scenarios [8–10], such as sustainable transport, considering different transportation product demands and coordinating with passenger transport. Chen et al. studied the integrated optimization of the selection of transfer stations and train schedule in the HSR express network [11].

Note that, it is necessary to conduct the operations as train reception and departure, loading and unloading at specific hubs for HSR freight trainsets. Such conditions are not currently available at high-speed passenger stations. Thus, a crucial aspect of the expansion of HSR freight is the construction of hubs outfitted with the appropriate operational conditions. In other words, the foundation of HSR freight development is the establishment of the HSR freight hub network. The design of a hierarchical and location scheme for high-speed rail freight transportation hubs has become a necessary decision for the development of the HSR freight transportation.

The collection and distribution between the hub and the customer are undertaken by road transport. The mode of operation resembles the movement of goods through a hub-and-spoke network. The hub location problem with hub-and-spoke networks and the HSR freight hub location are related.

Hub-and-spoke network is applied for passenger and freight airlines, express shipment and cargo delivery [12]. The hub location problem (HLP) considers the design of hub networks by selecting a set of nodes to locate hubs, activating a set of links, and routing commodities through the network while optimizing a cost-based (or service-based) objective function [13]. Since O’kelly first established a quadratic integer programming model to solve the hub location problems [14], the HLP has been extensively studied by scholars in terms of hub allocation, hub capacity levels, etc.

Since optimal allocations are affected by hub locations and optimal hub locations are affected by allocation decisions, location and allocation problems must be considered together in designing hub networks [15]. Typical allocation strategies between hubs and non-hubs are set forth: single allocation and multiple allocation. For cargo transportation, the single allocation structure is typically used by integrating link flows between hubs and utilizing economies of scale to reduce costs [12]. According to the capacity limit of the hub or the links, the HLP usually considers capacitated constraints. See the related research results in [16–19]. The application model for cargo transportation typically needs to take the delivery time constraint, among other factors, into account in order to guarantee the service [20–23] As a result, a non-hub node should be assigned to a single hub in the HSR freight network, and the hub construction usually needs to take service level limits into account.

In a multi-level node logistics network, in order to guarantee that each segment can choose the most cost-effective transportation mode to complete transportation process, large-scale logistics companies usually operate a variety of transportation modes, including air, road and rail. The problem that logistics companies must combine a variety of transport modes in order to be efficient and effective is solved by the design of multimodal transport networks in the literature. Alumur et al. design the decisions of different transport modes for the multimodal hub location and hub network design [24]. Jointly considering transportation costs and travel times, the design of multimodal hub network with different types of delivery time promises is discussed by Alumur et al. [25]. Wang and Meng study the discrete intermodal transport network design for cargo transportation from the perspective of network planner [26]. Zhou et al. study a hierarchical multimodal hub and allocation problem for China railway express [27]. Mostert et al. propose a bi-objective mathematical formulation which takes into account economic and environmental objectives on a road and intermodal network with three modes of transport [28]. Considering different transport modes and capacity levels, mathematical models are formulated for multimodal transport network under uncertainty in [29–34]. Fotuhi and Huynh, Torkestani et al. Maiyar and Thakkar and Zhalechian et al. deal with the multi-period intermodal hub location problems under disruption risks [23, 35–38].

The HSR freight is a new logistics modal with significant development potential that depends on the ongoing enhancement of the high-speed railway network. Although the HSR freight transportation has a relatively short operating history and there are still many issues to be studied, numerous scholars have explored issues related to freight transportation network design and hub location, establishing a sound theoretical foundation and providing strong theoretical support for the development of high-speed rail freight transportation networks. Currently, high-speed rail in China is mainly used for passenger transportation. However, as the high-speed rail network in China continues to expand and mature, it is important to fully utilize the advantages of high-speed rail in terms of speed, cost, and transport capacity, seize the opportunities to develop fast freight transportation services, establish a scientifically sound high-speed rail freight transportation system, open up new markets for fast freight transportation, and improve the overall economic benefits of railways. This has become a pressing and primary issue that needs to be addressed. Different from the current research focus, the paper’s contribution is defining the HSR freight hub location problem by the classical hub location model featuring as single allocation rule and configuring varying levels of hubs, as to provide a strategy for designing the HSR freight hub network. We develop mixed integer programming (MIP) model and efficient algorithms based on the conventional hub location problem and the theory of multimodal transport network design, taking into account the actual circumstances of the HSR freight hub location. The essence of the proposed problem is to obtain the HSR freight hub location scheme based on the existing HSR network facilities.

The format of this paper is as follows. The function of hubs and the HSR freight network are described in **Section 2**. **Section 3** studies the strategy of logistics hub location for HSR freight transport with road-rail intermodal transport network. A MIP model for the single-allocation capacitated hub location problem expanded to two transport modes is presented. **Section 4** provides a hybrid heuristic algorithm for this problem. The numerical experiments in **Section 5** obtain the hub location schemes and demonstrate the efficiency of the algorithm. The paper concludes with some observations and suggestions in **Section 6**.

## 2. Problem description

**Fig 1:**
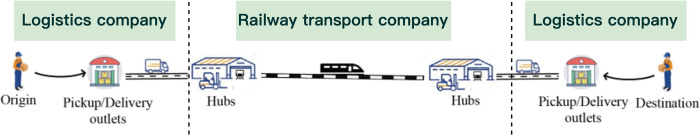
The function of the road-rail intermodal transport system.

In the logistics network, hub nodes and non-hub nodes are connected by road transport. Non-hub nodes are outlets operated by logistics companies that are also responsible for collecting and distributing customers’ parcels. Railway transportation corporations manage hubs and formulate the HSR freight train organization plan for cargo transportation. There is no existing HSR freight hub in the existing HSR network facilities, so it is necessary to build HSR freight hubs and design HSR freight hub network. The hubs handle the receiving, dispatching, loading and unloading of the HSR freight trains as well as warehousing. The function of the road-rail intermodal transport system is depicted in **Fig 1**.

**Fig 2:**
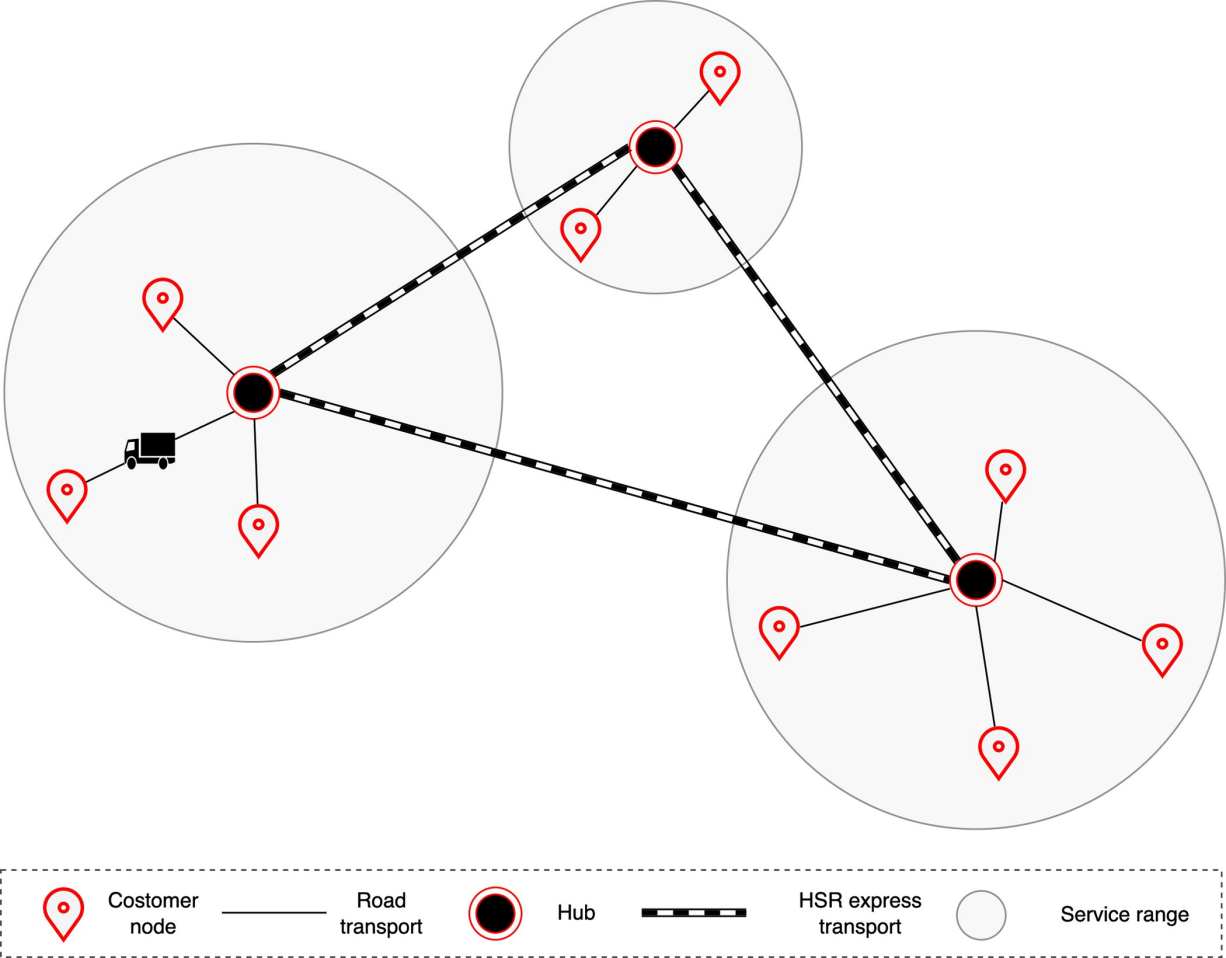
The hub-and-spoke network for HSR freight.

The HSR network is described as a hub-and-spoke network. Goods are transported in this network by a variety of modes (HSR freight trains, direct road transport and road–rail intermodal transport). In terms of road–rail intermodal transport, goods are transported by road from the origin to the HSR freight hub, then moved to another hub via HSR freight train, finally moved to the destination by road transport. The hub-and-spoke network for HSR freight is shown in **Fig 2**.

There are two different types of nodes in the HSR freight network: customer nodes and HSR freight hubs. OD transport demands of cargo are generated by customer nodes. The HSR freight hub is capable of reception and departure for HSR freight trains. Different levels of HSR freight hubs differ in their construction costs, capacities and service radiuses. With the designed hub-and-spoke network, the essence of the proposed problem is to obtain the HSR freight hub location scheme based on the existing HSR network facilities, which features as single allocation rule and configures varying levels of hubs to optimize the levels of hubs, customer allocation and cargo routing.

**Fig 3:**
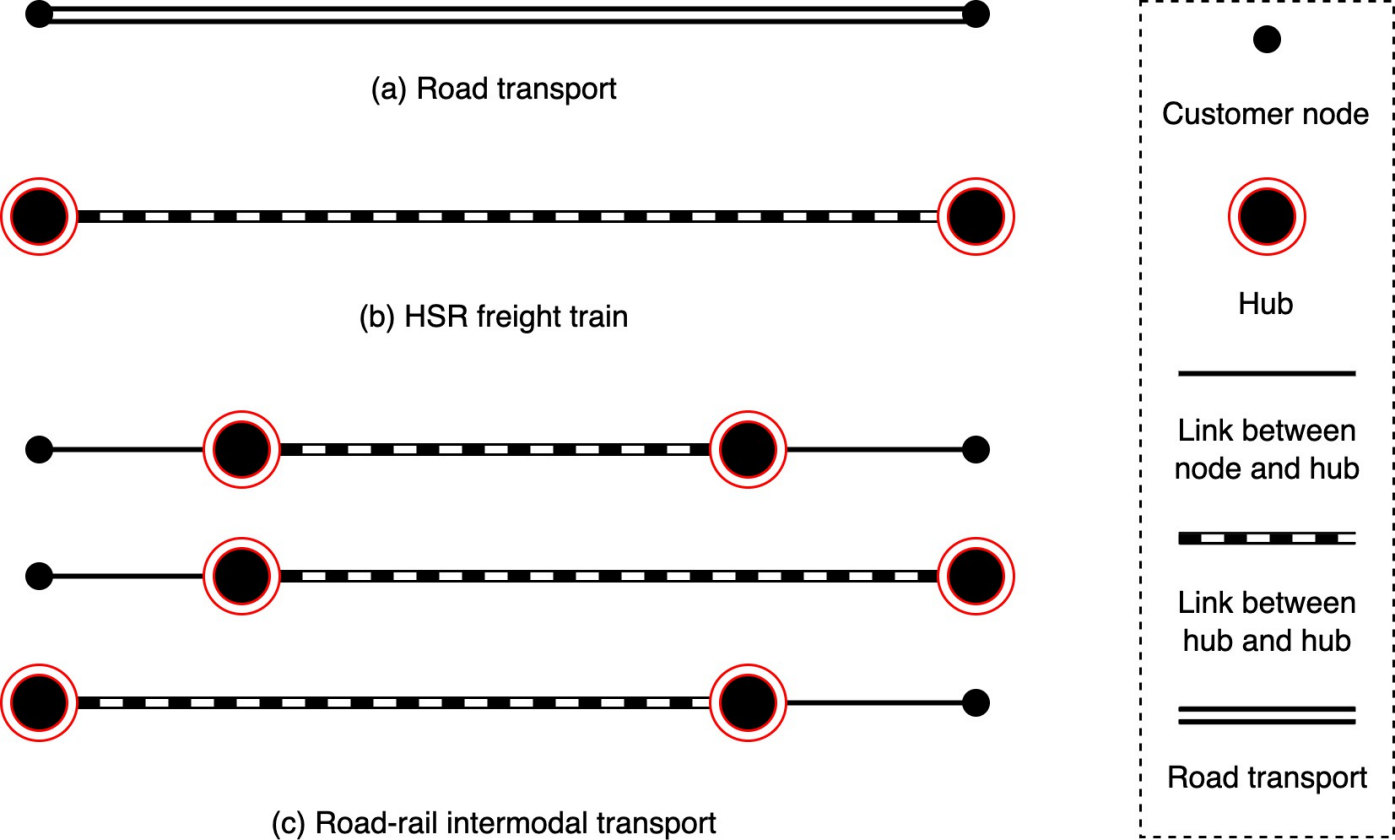
The transport modes in the HSR freight network.

Therefore, the problem is divided into three subproblems: location, allocation and mode selection. (1) Location subproblem: the decision on the selection of a group of hubs from a set of alternative hubs, varying in different levels with different construction costs, capacities and service radius. (2) Allocation subproblem: the decision on the assignment of customer nodes to a single hub. The customer node must to be situated within the hub’s service radius. (3) Mode selection subproblem: the decision on the transport mode for the cargo flows. Road transport (Mode 1) describes that the goods are delivered by road from the origin to the destination. Road–rail intermodal transport (Mode 2) is the movement of goods from the origin to the destination is completed by the HSR freight train including the situation where the starting point and the ending point are hubs. Parcels are transported by road to the HSR freight hubs and HSR freight trains can only operate on links between HSR freight hubs. The transportation modes are shown in **Fig 3**. The mode that an OD pair pass more than two hubs will greatly increase the handling time of parcels, reduce the transport efficiency, and can’t give full play to the advantages of HSR freight. Therefore, an OD pair pass two hubs at most.

Let *N* be the set of customer nodes in the network, *K* be the set of alternative hubs, *S* be the hub’s levels. *K*^*s*^ is the set of alternative hubs at different levels. The alternative hubs are selected from the set of customer nodes (*K*⊆*N*) and *S* = {1,2,…,*l*}. For each *k*∈*K*^*s*^, with a capacity *U*^*s*^ and a service radius *R*^*s*^ corresponding to level *s*, hence varies in construction cost *F*^*s*^. The hub’s handling capacity is restricted by *U*^*s*^, meaning that the amount of cargo turnover it engages in cannot exceed *U*^*s*^. *R*^*s*^ limits the range of customer nodes served by the hub in terms of distance.

The hub-and-spoke network is characterized by an inter-hub link (*k*−*m*) clustering effect that results in reduced network transport costs due to economies of scale. The discount cost can be fixed, linear, or nonlinear. The unit transport cost between hubs will remain steady in a hub-and-spoke network with high traffic levels [39]. We apply a fixed discount factor *α* in our study.

## 3. Mathematical formulations

In this section, we describe the HLP for HSR freight by a mixed integer programming model. The objective functions of HLPs are mainly cost-oriented, and few scholars consider time, carbon emissions and other aspects [40]. From the perspective of the overall national planning, the optimization objective of the model is to minimize the total network cost. Total network costs include fixed costs and variable costs. The fixed cost includes the construction and operating costs which is determined by the number and level of the located hubs. The variable cost refers to the network transport cost, which is based on the flow of goods in the network and the unit transport cost.

Given that the hubs are fully interconnected, any pair of hubs (*k*−*m*) can be served by HSR freight train. The transport demand from origin node *o*(*i*)∈*N* to destination node *d*(*j*)∈*N* can be satisfied by the three modes in **Section 2**, but only one path needs to be routed.

We define binary variables $y_{k}^{s}$, *k*∈*K*^*s*^ and *s*∈*S*, equal to 1 only if the hub *k* is located with level *s*. Simultaneously binary variables *x*_*ikmj*_ and *x*_*ij*_, *k*, *m*∈*K*^*s*^ and *i*,*j*∈*N*, are introduced, equal to 1 when the transport demand from *i* to *j* is served by mode 3(the path is *i*−*k*−*m*−*j*) and mode 1(the path is *i*−*j*) respectively. Correspondingly, the integer variables *q*_*ikmj*_ and *q*_*ij*_ represent the actual flow of goods distributed to each of the two paths.

We summarize all the notations in **Table 1**.

**Table 1:** Notations in the model.

Sets:	
*N*	The set of customer nodes in the network
*K*	The set of alternative hubs
*K* ^*s*^	The set of alternative hubs at different levels
*S*	The set of level of hubs (*S* = {1,2,…,*l*})
**Decision variables:**	
$y_{k}^{s}$	1 if the hub *k* is located with level *s* and 0 otherwise (*k*∈*K*^*s*^, *s*∈*S*)
*a*_*ik*_	1 if the hub *k* is allocated to the node *s* and 0 otherwise (*k*∈*K*^*s*^, *i*∈*N*)
*x*_*ikmj*_	1 if flow from node *i* to node *j* routed via hub *k* and hub *m* and 0 otherwise (*i*,*j*∈*N*, *k*,*m*∈*K*^*s*^)
*x*_*ij*_	1 if flow from node *i* to node *j* routed by road and 0 otherwise
**Parameters:**	
*q*_*ikmj*_	Flow from node *i* to node *j* via hub *k* and hub
*q*_*ij*_	Flow from node *i* to node *j* routed by highway
*n*	Number of nodes
*d*_*ij*_	Distance from node *i* to node
$F_{k}^{s}$	Construction cost of hub *k* at level *s*
$c_{r}^{ij}$	The road transportation cost of goods per unit weight transported in the arc (*i*−*j*)
$c_{h}^{ij}$	The HSR freight transportation cost of goods per unit weight transported in the arc (*i*−*j*)
*c*_*k*_	Handling cost per unit flow via hub *k*
*q*_*k*_	Flow via hub *k*
*p*_*s*_	Maximum number of hubs at level *s*
*l*	Maximum level of the hubs
*Q*_*ij*_	Transport demand from node *i* to node *j*
*U* ^*s*^	Maximum capacity of hubs at level *s*
*R* ^*s*^	Service radius of hubs at level *s*
*u*_*km*_	Maximum capacity of link between hub *k* and hub *m*
*D*_*hmax*_	Maximum transport distance of HSR freight train
*D*_*hmin*_	Minimum transport distance of HSR freight train
$t_{ik}^{r}$	Delivery time of road transport from node *i* to hub *k*
$t_{km}^{h}$	Delivery time of HSR freight transport from hub *k* to hub *m*
$t_{k}^{p}$	Handling time of hub *k*
*T*	The maximum commitment time of delivery


$$\min\sum_{k}^{n}F_{k}^{s}y_{k}^{s}+\sum_{i}^{n}\sum_{j}^{n}\sum_{k}^{n}\sum_{m}^{n}q_{ikmj}x_{ikmj}(c_{r}^{ik}+\alpha c_{h}^{km}+c_{r}^{mj})+\sum_{i,j}^{n}c_{r}^{ij}q_{ij}x_{ij}+\sum_{k}^{n}c_{k}(\sum_{i,j,m}^{n}q_{ikmj}x_{ikmj}+\sum_{i,j,m}^{n}q_{imkj}x_{imkj})$$ (1)

*S*.*t*.

$$\sum_{s}y_{k}^{s}\leq 1,\forall k\in K^{s},s\in S$$ (2)

$$\sum_{k}y_{k}^{s}\leq p_{s},\;\forall k\in K^{s},s\in S$$ (3)

$$a_{ik}\leq \sum_{s}y_{k}^{s},\;\forall k\in K^{s},s\in S,i\in N$$ (4)

$$\sum_{k}a_{ik}\leq 1,\forall k\in K^{s},i\in N$$ (5)

$$\sum_{m}x_{ikmj}\leq a_{ik},\forall k,m\in K^{s},i,j\in N$$ (6)

$$\sum_{k}x_{ikmj}\leq a_{jm},\forall k,m\in K^{s},i,j\in N$$ (7)

$$x_{ij}+\sum_{k,m}x_{ikmj}=1,\forall k,m\in K^{s},i,j\in N$$ (8)

$$q_{ij}x_{ij}+\sum_{k,m}q_{ikmj}x_{ikmj}=Q_{ij},\forall k,m\in K^{s},i,j\in N$$ (9)

$$a_{ik}∙d_{ik}\leq \sum_{s}R^{s}∙y_{k}^{s},\forall k\in K^{s},i\in N,s\in S$$ (10)

$$\sum_{i,j,m}^{n}q_{ikmj}x_{ikmj}+\sum_{i,j,m}^{n}q_{imkj}x_{imkj}\leq \sum_{s}U^{s}∙y_{k}^{s},\forall k,m\in K^{s},i,j\in N,s\in S$$ (11)

$$\sum_{i,j}^{n}q_{ikmj}x_{ikmj}\leq u_{km},\forall k,m\in K^{s},i,j\in N$$ (12)

$$x_{ikmj}∙d_{km}\leq D_{hmax},\forall k,m\in K^{s},i,j\in N$$ (13)

$$x_{ikmj}∙D_{hmin}\leq d_{km},\forall k,m\in K^{s},i,j\in N$$ (14)

$$x_{ikmj}(t_{ik}^{r}+t_{km}^{h}+t_{jm}^{r}+t_{k}^{p}+t_{m}^{p})\leq T,\forall k,m\in K^{s},i,j\in N$$ (15)

$$y_{k}^{s}\in \{0,1\},\forall k\in K^{s},s\in S$$ (16)

$$a_{ik}\in \{0,1\},\forall k\in K^{s},i\in N$$ (17)

$$x_{ij}\in \{0,1\},\forall i,j\in N$$ (18)

$$x_{ikmj}\in \{0,1\},\forall k,m\in K^{s},i,j\in N$$ (19)

The objective function **(1)** minimizes the total costs including construction of hub facilities and transportation costs as well as handling costs of hubs. Construction costs usually refer to the construction costs of new hub facilities and the fixed operating costs after completion. Transportation cost refers to the cost incurred during the transportation of goods through one of the three transportation modes. And There will be certain handling costs incurred for goods at the hub as a result of loading and unloading. Constraint **(2)** ensures that a hub can only correspond to one level. Constraint **(3)** limits the open hubs amount. Constraint **(4)** the allocation of nodes to their corresponding hubs is single. Constraint **(5)** indicates that nodes can only be assigned to a single hub. Constraint **(6)** and **(7)** limit the flows served by mode 1 and mode 3 will be assigned to an open hub pair. Constraint **(8)** restricts that the flows can be served by one transportation mode only. Constraint **(9)** ensures that the transport demands in the network are all satisfied. Constraints **(10)** limit the service scope of the hub at several levels. Constraints **(11)** specifies the upper limit of the handling capacity for variable levels of hubs. Constraints **(12)** limit the capacity of inter-hub links. Constraints **(13)** and **(14)** set the upper and lower limits of service distances for HSR freight respectively. Constraint **(15)** guarantees that the road-rail intermodal transport needs to be completed within the maximum commitment time. Constraints **(16)-(19)** are constraints on the values of the decision variables.

## 4. Heuristic algorithms

The inter-modal transport hub location problem with single-allocation capacitated constraints proves to be an NP-hard problem [41]. We propose a hybrid heuristic algorithm to solve this problem that combines variable neighbourhood descent and tabu search algorithm efficiently. In order to improve the efficiency of the algorithm, we design a greedy strategy-based construction approach for the initial hub location solution. A reasonable solution strategy is also provided for the allocation and mode selection subproblems.

### 4.1. The construction of initial hub location solution based on the greedy strategy

We construct the initial hub location solution based on greedy strategy. Since the construction costs of hubs are positively correlated with the number of open hubs, the network transport costs will be significantly decreased with the growth of economies of scale in inter-hub links. The main idea of the construction approach is to allocate each customer node to the alternative hubs that is nearest to them within their service ranges, resulting in obtaining the open hub set. The number of open hubs is then reduced through demand consolidation, until a better feasible solution is iterated.

**Algorithm 1: A greedy constructive method.**

allocate hub *k* to each node *i*

**while** *k*_*l*_≥*p* **do**

      **for** all *k*_*op*_∈ℒ **do**

            convert *k*_*op*_ to *k*_*cl*_

            allocate hub *k* to each node *i*

            **if** ℱ(ℒ′,A′)<ℱ(ℒ,A) **do**

                  update ℒ,A

            **end if**

      **end for**

      **if *k***_***l***_ **= *k***_***l***_ **do**

            break

      **end if**

*k*_*l*_ = *k*_*l*_−1

**end while**

Let ℱ(ℒ,A) denote the objective function, that is the total network cost associated with the hub location decision ℒ and the allocation decision A. ℱ(ℒ′,A′) is obtained by closing an open hub (convert *k*_*op*_ to *k*_*cl*_) in ℒ and reallocating when the number of open hubs *k*_*l*_ in ℒ is not less than the minimum *p*. Update ℒ and A when the objective function is optimized, otherwise terminate the procedure. The greedy constructive method is shown in **Algorithm 1**.

First, the initial hub location scheme is obtained once all the demands have been assigned. Then we consider that the objective function can be optimized by lowering the number of open hubs as much as possible. Therefore, it is thought that a better feasible solution has been obtained with no changes in *k*_*l*_, which is set as one of the end conditions of the procedure. As a result, we obtain the initial hub location solution based on greedy strategy.

### 4.2. Solving the allocation and mode selection subproblems

**Fig 4:**
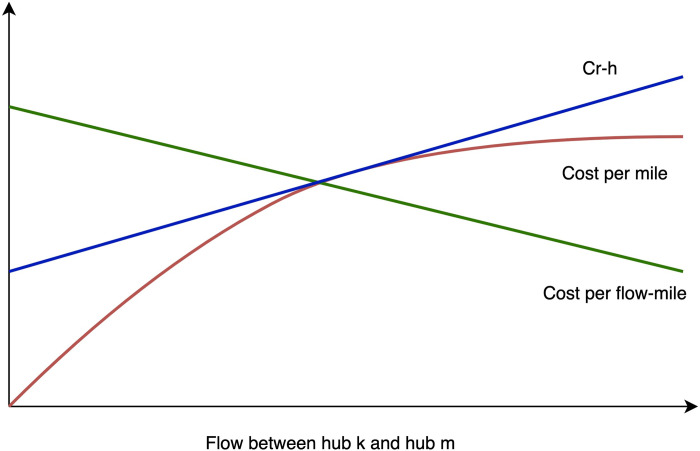
The economic scale effect of the hub.

In the case where the hub location solution is given, it’s also a problem of complexity *O*(*n*^4^+*n*^2^) to solve the allocation and mode selection subproblems. To improve the efficiency of the hybrid heuristic algorithm, we still apply the greedy strategy to present the solution of the allocation and mode selection subproblems. As the cargo flow allocated to inter-hub links rises, the objective function has a tendency to optimize with a specific open-hub scheme. This is because inter-hub links produce economies of scale, which greatly lowers the unit transport costs of the links. As can be observed in **Fig 4**, the hub-and-spoke network is characterized by the connection between hubs resulting in an effect known as economies of scale, which causes the transport costs between hubs to rise at a decreasing rate and finally tends to be stable [42].

The customer node is single allocated to the hub in this problem. In actuality, the hub also performs as a sort of consolidation spot for HSR freight work. Since the lower-level nodes and hubs are typically fixed matches, it is simpler to execute the distribution tasks precisely. As a result, with a single allocation rule and a stable unit transportation cost, the allocation of nodes depends on the following two criteria: (1) Nodes will be allocated to open hubs that are closer in priority, provided they are located within the service radius of the hub. (2) The hubs are also limited by their own handling capacity. Hubs are not allowed to handle excessive flows in the network. The remaining capacity of the hub needs to be taken into account.

We define an inequality with respect to the ratio of distance to radius as well as the ratio between flow and capacity:

$$V_{ik}=\frac{q_{i}}{U_{k}}∙\frac{d_{ik}}{R_{k}}\leq V_{p}$$ (20)

**Fig 5:**
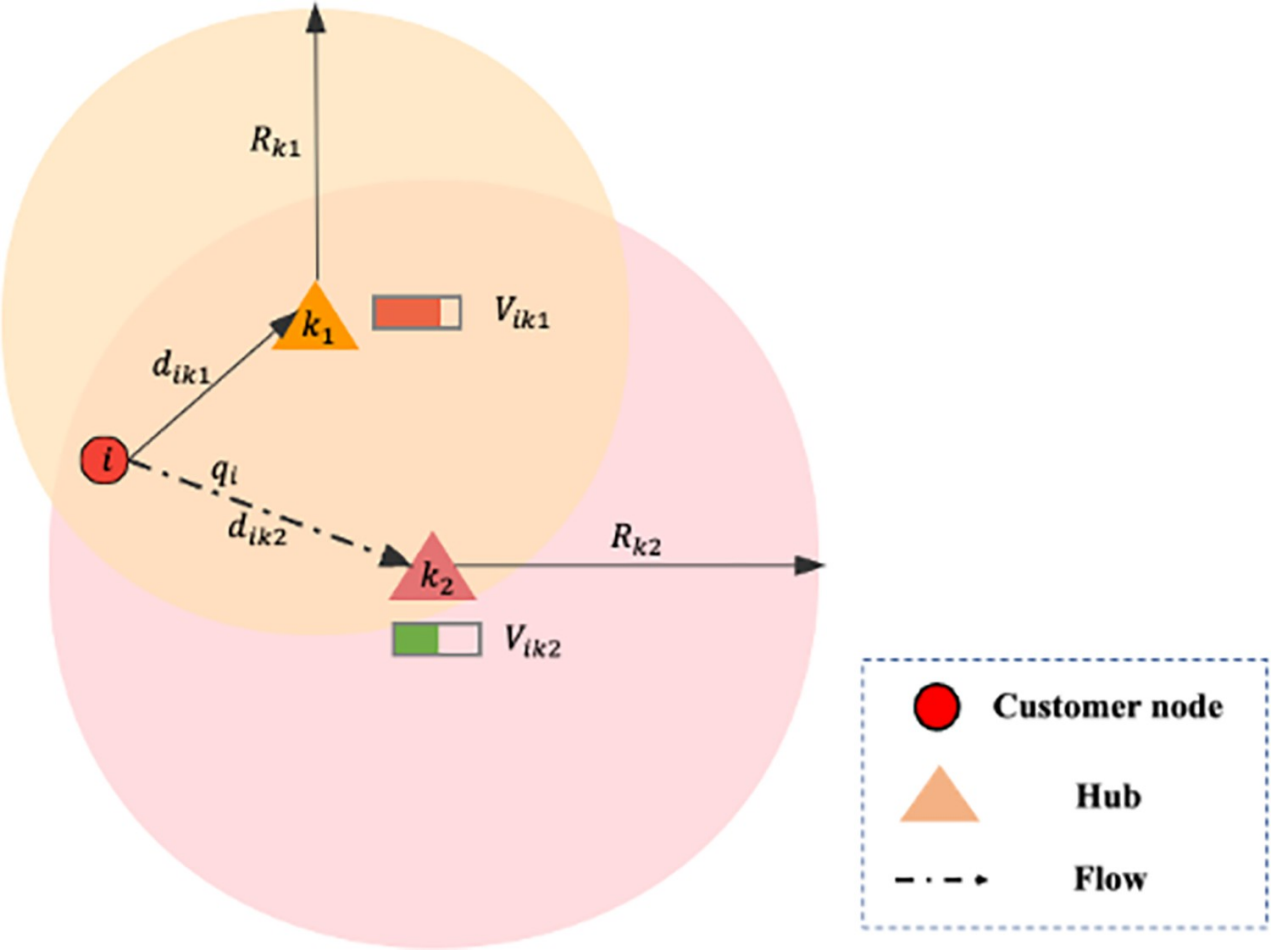
An example of allocation for node *i*.

The delivery and distribution flows of node *i* comprise its total demand, denoted as *q*_*i*_. The allocation of node *i* to hub *k* will potentially violate the capacity constraint when the ratio of *q*_*i*_ to *U*_*k*_ is considerable. It gains priority that node *i* allocated to a hub that is comparatively farther distant as shown in **Fig 5**.

The control degree of the node *i* assigned to hub *k* is indicated by the new variable *V*_*ik*_. With the increase of *V*_*ik*_, there is a lower priority for node *i* allocated to hub *k*. For each potential hub of node *i* that satisfies the service radius constraint, *V*_*ik*_ is determined. The allocation solution for node *i* is chosen as the hub k satisfying *V*_*ik*_≤*V*_*p*_ and having the smallest value out of all the outcomes in *V*_*ik*_. Here we set a new parameter *V*_*p*_, known as the control parameter, for which value is obtained through numerical experiments in **Section 5.2**.

**Fig 6:**
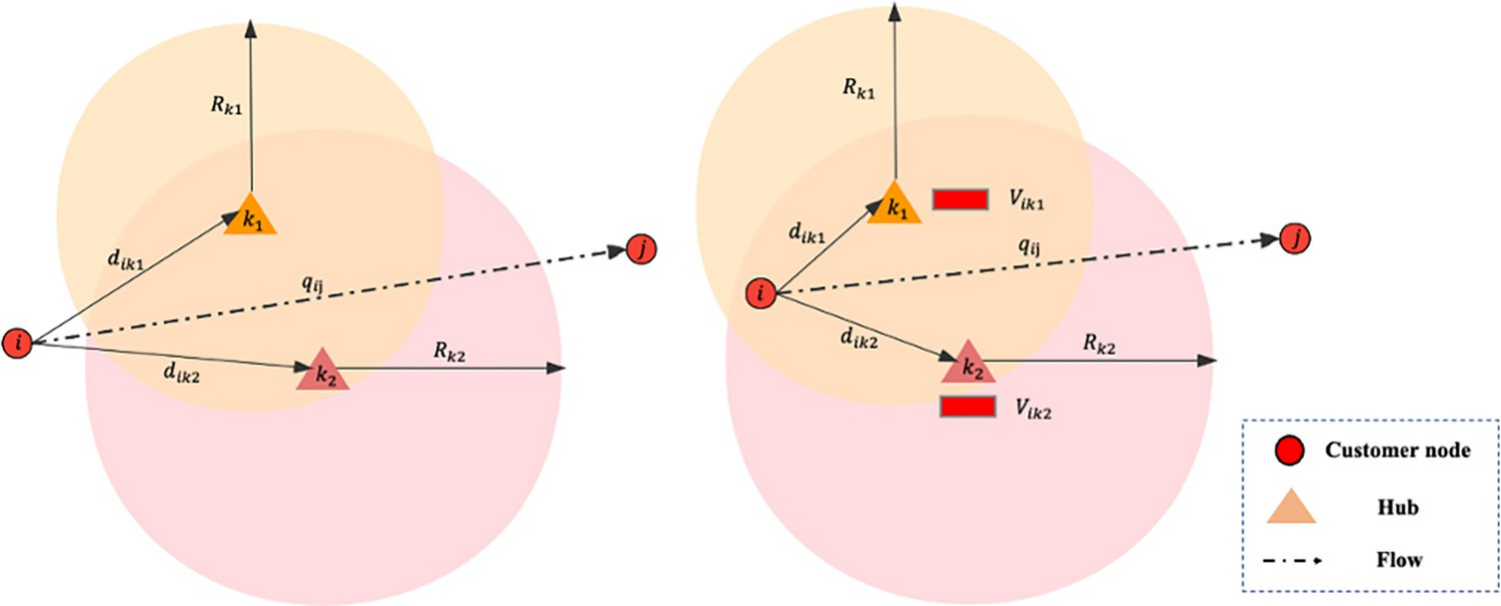
An example of node *i* not allocated to any hub.

There are two situations as follows: (1) Node *i* is not located within the service radius of any open hub. (2) For all hub *k* belongs to the set of candidate open hub for node *i*, *V*_*ik*_>*V*_*p*_. Node *i* will not be assigned to any hub if either of the aforementioned circumstances holds true. All flows of O-D pairs at node *i* will be served via direct road transport mode as shown in **Fig 6**.

We designed a specialized procedure for resolving the allocation and mode selection subproblems in **Algorithm 2** after the allocation principles were established. We initially choose open hubs *k*_*op*_ that satisfy the service radius constraint for each node *i* to be added to the candidate hub set X_*i*_ based on the hub location solution X_*location*_. Node *i* is then assigned to hub k, which is found to satisfy *V*_*ik*_≤*V*_*p*_ and have the minimum value of *V*_*ik*_ in the set X_*i*_. Otherwise, no hub is assigned to node *i*. The O-D flow allocation and transport mode scheme for all customer nodes in the network are obtained automatically.

Each O-D pair is evaluated after allocation to ensure if it satisfies the distance-time and time-of-service deadlines for the HSR freight service. If both conditions are met, *f*_*ikmj*_ and *f*_*ij*_ are calculated, and the flow is then assigned to the path with minimum *f*. Otherwise, the path designated for road transport receives the flow.

**Algorithm 2: A procedure for solving allocation and mode selection subproblem.**

**for** all *i*∈*N* **do**

 **for** all *k*_*op*_∈X_*location*_ **do**

**if *d*_*ik*_≤*R*_*k*_ do**

X_*i*_ ← add *k*_*op*_

          **end if**

      **end for**

      **for** all *k*∈X_*i*_ **do**

            calculate *V*_*ik*_

      **end for**

      find *k* with min{*V*_*ik*_}

      **if** min{*V*_*ik*_}≤*V*_*p*_ **do**

            allocate *i* to *k*

      **else**

            X_*i*_ = ∅

      **end if**

**end for**

**for** all *i*∈*N* **do**

      **for *j*∈*N* do**

**if X_*i*_ = ∅** or **X_*j*_ = ∅ do**

                *q*_*ij*_ = *Q*_*ij*_

           **else**

**if** *D*_*hmin*_≤*d*_*km*_≤*D*_*hmax*_ and *t*_*rr*_≤*T* **do**

     **if *f***_***ikmj***_**≤*f***_***ij***_ **do**

                         *q*_*ikmj*_ = *Q*_*ij*_

                        **else**

*q*_*ij*_ = *Q*_*ij*_

 **end if**

                  **else**

                        *q*_*ij*_ = *Q*_*ij*_

        **end if**

**end if**

**end for**

**end for**

### 4.3. A hybrid heuristic algorithm based on variable neighbourhood descent and tabu search

We propose a hybrid heuristic algorithm in this section, combining the advantages of tabu search and variable neighbourhood descent.

#### 4.3.1. Tabu search

The Tabu Search (TS) algorithm was presented by Glover [43]. It is characterized by the proposed methods to improve the method of being stuck in the local optimum during the search process. A sub-heuristic random search technique starts with a feasible initial solution and chooses a number of specified search directions (moves) as a trial, choosing the move that results in the greatest change in the value of a given objective function. A flexible "memory" mechanism is utilized in TS to record and select the optimization process that has been carried out and to guide the next phase in the search in order to prevent being stuck in a local optimum.

The hybrid algorithm we propose next will introduce the rules as tabu list and aspiration criterion of TS, the objects in tabu list are hub location solutions. The tabu length is set as $\sqrt{n}$. Neighbourhood structure is proposed in **Section 4.3.2**. If the evaluation value of a solution is higher than any best candidate solution, it can be released.

#### 4.3.2 Variable neighbourhood descent

Hansen and Mladenović devised the solution refining technique known as Variable Neighbourhood Descent (VND), which performs alternating searches in the solution space while methodically taking the neighbourhood structure into account [44].

**Fig 7:**
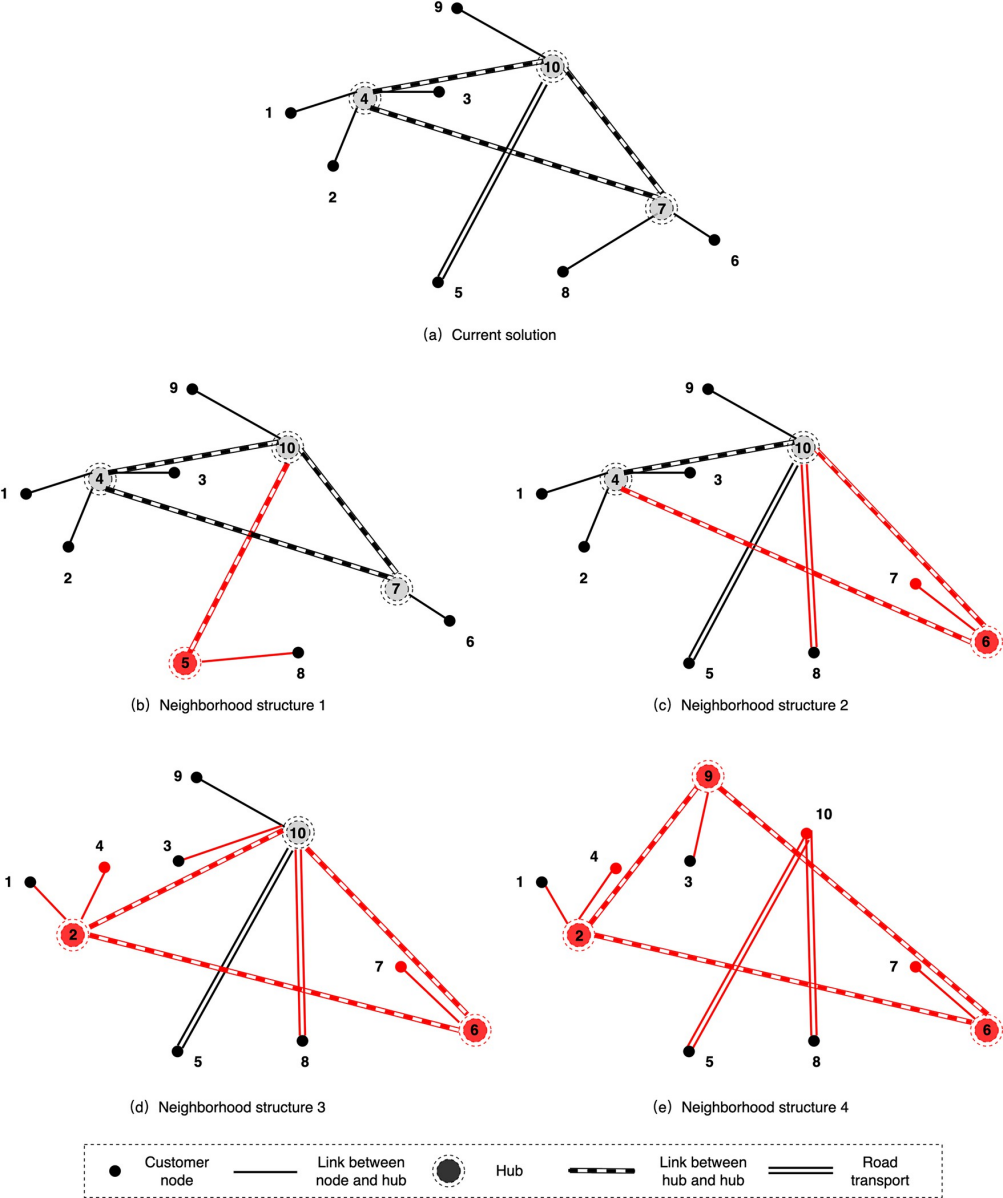
The neighbourhood structure in VND.

We create the following neighbourhood structures for the hub location solutions as shown in **Fig 7** since effective techniques for the allocation and mode selection subproblems have been provided in **Section 5.2**.

*N*_1_: The set of all feasible solutions formed through the operation on the current hub location solution by opening or closing a hub. The operation seeks to alter the number of open hubs and significantly changes the function value.

*N*_2_: The set of all feasible solutions formed through performing a hub swap operation on the current hub location solution. Only one pair of hubs can be switched at once, one from open hubs and one from closed hubs. In other words, the operation has no effect on the quantity of open hubs.

*N*_3_: The set of feasible solutions formed by conducting a hub swapping operation on the current hub location solution using a random number generator. We set a random integer, *γ*∈{2, *k*_ℒ_} with the aim of regulating the neighbourhood’s size. The upper bound of *γ*, *k*_ℒ_ is the number of hubs that can be exchanged currently and the equation is:

$$k_{L}=min\{k_{op},k_{cl}\}$$ (21)

In fact, the value of *k*_ℒ_ is equal to the minimum of the current number of open hubs, *k*_*op*_, and the number of closed hubs, *k*_*cl*_.

*N*_4_: The set of all feasible solutions that perform the same operation as *N*_3_. However, the number of exchange hubs takes all values in the set {2, *k*_ℒ_} except *γ*.

Due to the high construction costs, the operation in *N*_1_ will be the direction with the greatest degree of adjustment to the value of the objective function. In contrast, the search in *N*_2_, *N*_3_ and *N*_4_ is carried out with a fixed number of open hubs. We limit the number of possible solutions for each neighbourhood to a maximum of 200, in order to regulate the size of the neighbourhood.

#### 4.3.3 A hybrid heuristic algorithm

**Fig 8:**
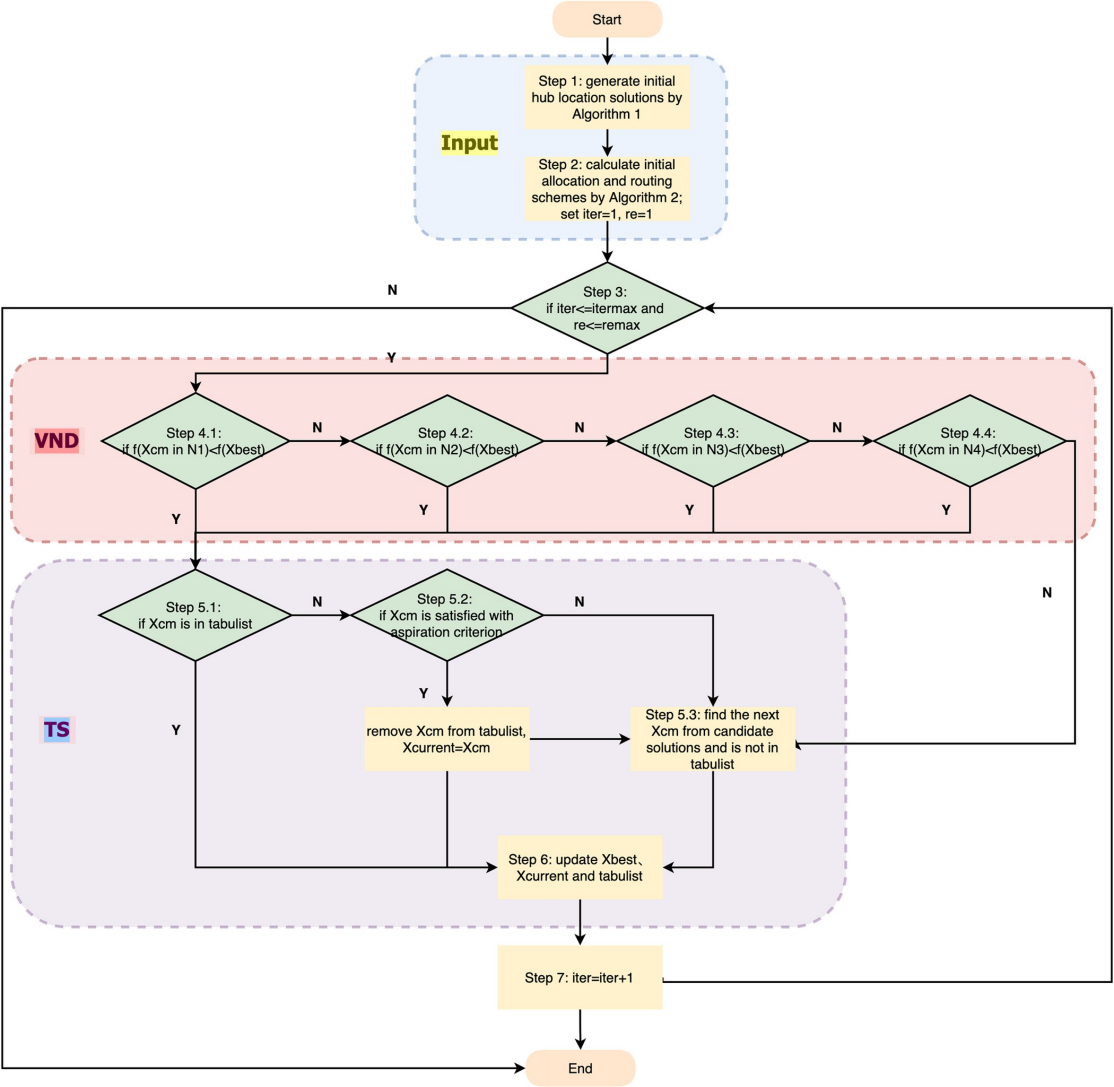
The structure of VND-TS.

Our hybrid heuristic algorithm called VND-TS is an extension of the local search algorithm. The main body adopts the structure of TS and the local search section applies the principles of VND. Although it is demonstrated that the VND algorithm is shown to have a tendency to fall into local optima, it has a significant ability to climb hills [45]. A receiving criterion of TS is introduced to prevent the search from trapping into local optimum as shown in **Fig 8**.

The specific procedure of the algorithm is as follows:

**Step 1:** Generate a feasible solution for initial hub location solution by **Algorithm 1**, and go to **Step 2**;
**Step 2**: Generate the corresponding allocation and transportation mode scheme using **Algorithm 2** according to the initial hub location solution and go to **Step 3**; set the initial scheme as the *X*_*current*_ and *X*_*best*_; set *iter* = 1, *re* = 0;
**Step 3**: Go to **Step 4.1** if *iter*< = *itermax* and *re*> = *remax*, otherwise output results;
**Step 4.1**: Enter the optimization search step of the algorithm, search for all feasible solutions in the neighbourhood *N*_1_ and calculate the function values. If the optimal candidate solution *X*_*cm*_<*X*_*current*_ in *N*_1_, go to **Step5.1**, otherwise go to **Step 4.2**;
**Step 4.2**: Search for all feasible solutions in the neighbourhood *N*_2_ and calculate the function values. If *X*_*cm*_<*X*_*current*_ in *N*_2_, go to **Step 5.1**, otherwise go to **Step 4.3**;
**Step 4.3**: Search for all feasible solutions in the neighbourhood *N*_3_ and calculate the function values. If *X*_*cm*_<*X*_*current*_ in *N*_3_, go to **Step 5.1**, otherwise go to **Step 4.4**;
**Step 4.4**: Search for all feasible solutions in the neighbourhood *N*_4_ and calculate the function values. If *X*_*cm*_<*X*_*current*_ in *N*_4_, go to **Step 5.1**, otherwise *re* = *re*+1 then go to **Step 5.3**;
**Step 5.1**: If *X*_*cm*_ is in tabu list, go to **Step 5.2**, otherwise go to **Step 6**;
**Step 5.2**: If *X*_*cm*_ is satisfied with aspiration criterion, release it then go to **Step 6**, otherwise go to **Step 5.3**;
**Step 5.3**: Find the next *X*_*cm*_ in candidate solution set which is not in tabu list, go to **Step 6**;
**Step 6**: Update the current solution and the tabu list, release the solutions, which tabu length is 0, go to **Step 7**;
**Step 7**: *iter* = *iter*+1, return **Step 3**.

## 5. Numerical experiments

The test instances are generated using the real data from cities’ express volumes in China. The network and O-D demands are given in **Section 5.1**. Next, the parameters are tested and the proposed hybrid heuristic method resolves the cases in **Section 5.2**. In **section 5.3**, the performance of our hybrid heuristic algorithm is then contrasted with that of Gurobi and other heuristic algorithms.

### 5.1. The network and data generation

**Fig 9:**
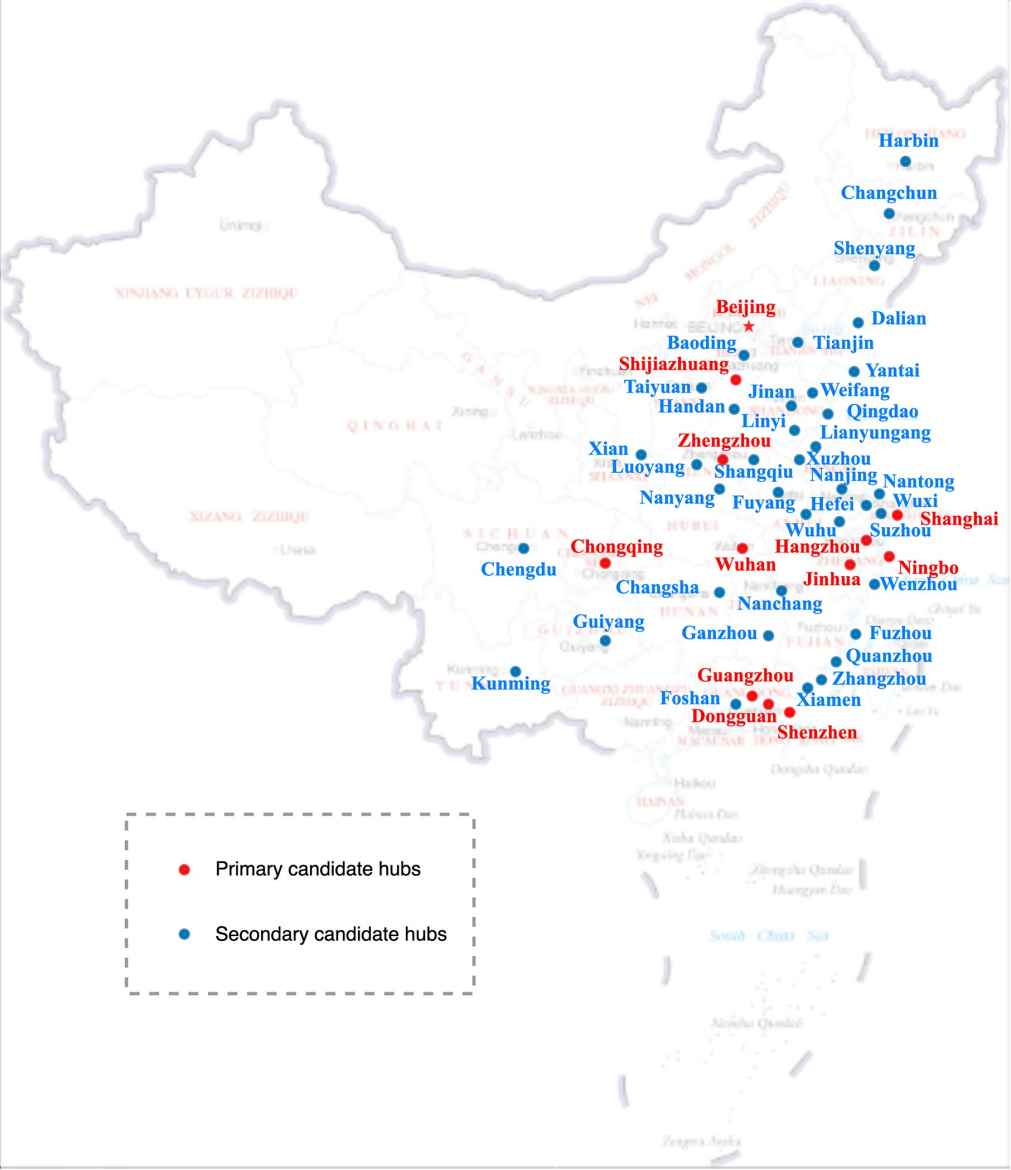
The instance network in China.

Based on the top 50 Chinese cities in terms of express volume in 2020, we forecast the average daily express volume in 2025 referring to [7], and obtain the O-D demands using a gravity model. We divide the cities in advance into two levels of candidate sets by clustering analysis of each city’s economic, demographic and logistic elements. The instance network is shown in **Fig 9**.

### 5.2. The network configurations with different parameters

**Fig 10:**
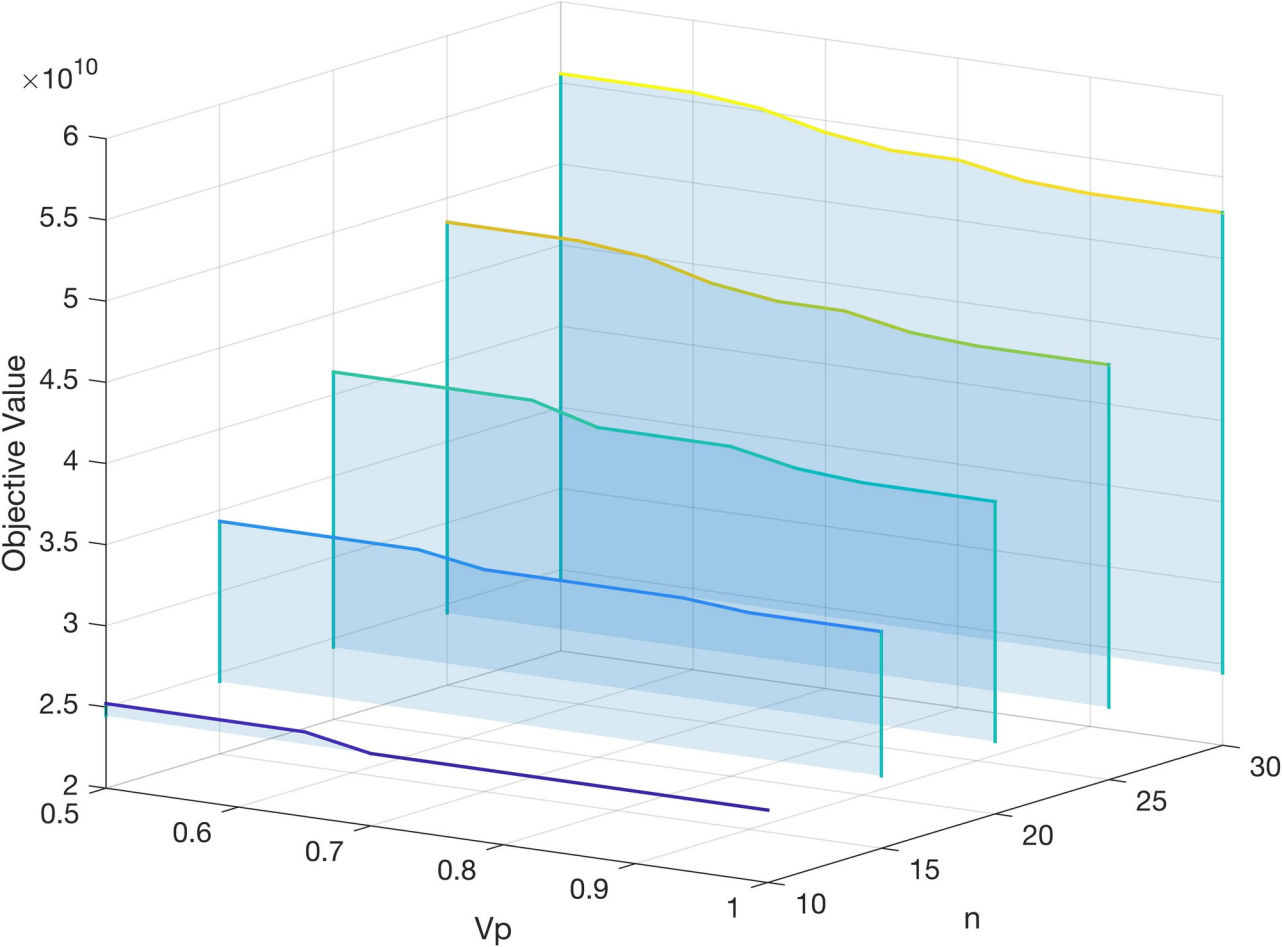
The performance of *V*_*p*_ with various network scales.

Before solving the instances, it is important to ascertain the value of control parameter *V*_*p*_. The influence of *V*_*p*_ on the values of the objective function through numerical experiments and satisfactory values of the objective function would be obtained based on the analysis results. Here we have experimented with various network scales. **Fig 10** demonstrates that the value of *V*_*p*_ rises with the network size and eventually leans to 0.9. The objective function for the network with a fixed number of nodes optimizes as *V*_*p*_ increases. Therefore, when solving the actual hub location scheme, we set this parameter to 0.9.

**Fig 11:**
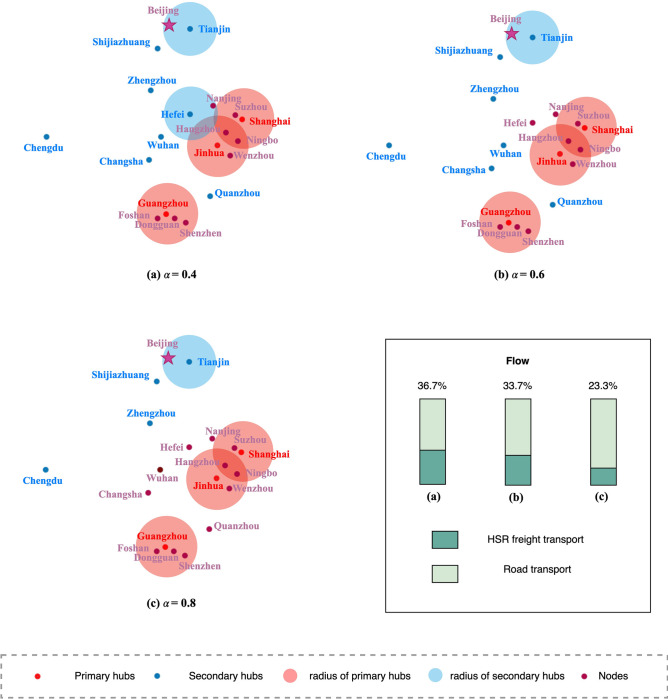
Network configurations with *α*∈{0.4,0.6,0.8}.

We experimented with instances of 20 nodes to analyze parameters for their effects on hub network configuration. The hub location schemes for the economies of scale factors of 0.4, 0.6, and 0.8 are shown in **Fig 11**, which shows the locations of hubs, connections in the hub network and the allocations of non-hub nodes to hubs. It can be seen that the hub-and-spoke network configuration is significantly impacted by economies of scale factor. The selection of the three primary hubs (Shanghai, Jinhua, and Guangzhou) remained unchanged due to their ability to cover a large number of nodes and their high demand for processing capacity, which was not affected by economies of scale. Due to the reduction of the economies of scale factor, the hub location scheme resulted in an increase in the number of secondary hubs. This is because increasing the number of hubs allows more parcels to be transported through road-rail transportation. Therefore, the flow of HSR freight transport gradually increases while the proportion of road transport decreases. This is consistent with the principle of the hub-and-spoke network, and controlling the cost of channels and the number of hubs can take advantage of the network structure to reduce the overall network cost.

Three levels of construction cost are set to evaluate its impact on the network configuration considering that the construction cost of the hub is hard to define. We test the outcomes of various economies of scale coefficients in a sample network of 20 nodes as well. The main conclusion in **Table 2** is that when the cost of opening the hub is at a high level, the scale economy coefficient affects the hub opening scheme more. The flow of intermodal transport is dramatically decreased once the hub link scale is not economical. The hub location scheme for primary hubs in the test cases was not affected by the opening costs of the three levels of hubs. This is because primary hubs have a larger coverage area and stronger processing capabilities, making them more irreplaceable in the network. Higher opening costs have more influence on the location of secondary hubs. However, when the hub opening costs decrease to a low level, the location results for secondary hubs become less sensitive, and the network configuration tends to be stable.

**Table 2:** Test with three levels of construction cost.

*F*_*k*_	*α*	Open primary hubs	Open secondary hubs	Proportion of intermodal flow
**High**	0.4	SH、JH、GZ	TJ、SJZ、ZZ、HF、CD、WH、CC、QZ	36.7%
	0.6	SH、JH、GZ	TJ、SJZ、ZZ、CD、WH、CC、QZ	33.7%
	0.8	SH、JH、GZ	TJ、SJZ、ZZ、CD	23.3%
**Med**	0.4	SH、JH、GZ	BJ、QZ、CD、WZ、SJZ、ZZ、WH、CS、HF	37.5%
	0.6	SH、JH、GZ	TJ、QZ、CD、WZ、SJZ、ZZ、WH、CS、HF	36.7%
	0.8	SH、JH、GZ	TJ、QZ、CD、SJZ、ZZ、WH、CS、HF	34.3%
**Low**	0.4	SH、JH、GZ	HZ、BJ、DG、QZ、CD、WZ、SJZ、ZZ、WH、CS、TJ、HF	37.4%
	0.6	SH、JH、GZ	HZ、BJ、QZ、CD、WZ、SJZ、ZZ、WH、CS、TJ、HF	36.8%
	0.8	SH、JH、GZ	HZ、QZ、CD、WZ、SJZ、ZZ、WH、CS、TJ、HF	36.1%

Note: the city names are represented by their initials.

In any case, lower channel costs and lower hub construction costs can make it easier for decision-makers to develop high-speed rail freight network infrastructure plans. Intuitively, nodes located in high-demand freight cities are more suitable to be selected as primary hubs. Although the amount of freight that can be transported through road-rail transportation in the high-speed rail freight network is limited by construction and channel costs, this approach is more in line with sustainable development strategies and can help reduce the overall cost of the freight system.

### 5.3. Comparison of algorithms

We study the single-allocation HLP for the rail-road intermodal transport at multiple capacity and service levels. The proposed mathematical model can apply Gurobi to solve small-scale instances. On the other hand, we examine a variety of heuristic algorithms performance on solving large-scale instances, and the computational results show that our hybrid heuristic algorithm is more efficient. It can be seen in **Table 3** that the best comprehensive performance is provided by VND-TS. In contrast to Gurobi and other heuristic algorithms, VND-TS offers the lowest average computation time. The maximum gap with the global optimal solution is 2.48%, and no more than 1% of the average gap.

**Table 3:** The experiment result comparison of algorithms.

Number of nodes	Number of candidate hubs		Gurobi			GA			TS			VND			VND-TS		
	P1	P2	Value (10^10^)	Gap (%)	Time (s)	Value (10^10^)	Gap (%)	Time (s)	Value (10^10^)	Gap (%)	Time (s)	Value (10^10^)	Gap (%)	Time (s)	Value (10^10^)	Gap	Time (s)
10	2	8	2.3935	0	2.36	2.3935	0	10.2	2.3935	0.00	1.57	2.3935	0	7.76	2.3935	0.00	3.17
	4	6	2.4435	0	3.48	2.4459	0.10	11.41	2.4459	0.10	1.72	2.4459	0.10	7.57	2.4459	**0.10**	2.94
15	2	13	3.2830	0	12.08	3.283	0	22.6	3.3052	0.68	3.18	3.283	0	20.71	3.283	**0.00**	**3.18**
	4	11	3.3330	0	5.82	3.3343	0.04	20.83	3.3837	1.52	3.49	3.3343	0.04	20.43	3.3343	**0.04**	**2.78**
20	4	16	3.9213	0	45.62	4.0462	3.19	30.23	4.0294	2.76	10.96	3.9233	0.05	23.09	3.9233	**0.05**	**8.99**
	6	14	3.9347	0	47.60	4.0953	4.08	36.88	4.0433	2.76	13.25	3.9363	0.04	22.47	3.9363	**0.04**	**8.105**
30	4	26	5.1528	0	494.32	5.6383	9.42	59.94	5.3432	3.70	32.19	5.2808	2.48	53.57	5.2808	**2.48**	**20.15**
	6	24	5.3279	0	488.42	5.8102	9.05	59.32	5.3432	0.29	32.38	5.3280	0	52.46	5.328	**0.00**	**25.81**
50	6	44	8.0479	0	60189.80	9.6205	19.54	773.53	8.7874	9.19	120.27	8.1514	1.29	196.71	8.0851	**0.46**	**100.69**
	12	38	8.1727	0	69510.56	8.603	5.27	784.33	9.204	12.62	160.71	8.2953	1.50	196.25	8.2292	**0.69**	**106.73**
Average			4.7817	0	12360.86	5.1175	5.21	196.60	5.0022	3.27	53.39	4.8149	0.51	65.13	4.8029	**0.36**	**31.79**

For the small-scale instances, Gurobi can find the exact solution in a short time. Additionally, four types of heuristic algorithms possess the ability to search near-optimal solutions rapidly. With the expansion of the network scale, solving time of Gurobi increases dramatically. A significant reduction in solving time is observed for these heuristic algorithms, but various characteristics of algorithms exist. Regarding their abilities to search solutions, VND and VND-TS perform the best and with the most stability. Particularly, VND-TS is comparatively better at escaping the local optimum. Both TS and VND-TS take less time to obtain a satisfactory solution, while TS gives high randomness and VND-TS exhibits higher stability and has a considerable advantage in the objective function values. Furthermore, the solution time of GA (Genetic Algorithm) is obviously not dominant and the average gap is higher than 5%.

## 6. Conclusion

The purposes of this paper are proposing the HSR freight hub location problem and transportation mode based on the sufficient resources of China’s high-speed rail network. We propose an application to locate the optimal configuration of road-rail intermodal transport hub for HSR freight transport and conduct cargo flow allocation with hub-and-spoke network. A single-allocation capacitated hub location model is proposed for this application to address the special limitations of HSR freight transport, such as transportation distance and commitment time on the basis of the traditional HLP formulations.

We propose a hybrid heuristic algorithm in order to solve this NP-hard problem on the actual network. The algorithm first designs the initial solution based on the greedy strategy and gives the program to obtain the allocation and mode selection scheme. The framework of the algorithm is then developed combining the benefits of VND and TS. This approach can be applied to plan a high-speed rail freight network based on anticipated freight demand. The gap between the exact solution and our hybrid heuristic approach in the instance tests is less than 1%. Its higher stability and efficiency compared to other heuristic algorithms verify the effectiveness of this approach.

The case results obtained by using the proposed model and algorithm to solve actual market cases can assist network designers in making hub location decisions for the high-speed rail freight network at a macro level while minimizing channel transportation costs and construction costs.

Further research may focus on considering uncertain transport demand. By considering such demand, the model can be applied to various scenarios, such as the peak demand for parcel transportation during shopping festivals. Network design based on fuzzy demands is a common problem in practical applications. In the next step we will construct robust planning models to formulate this problem in the future. In addition, by incorporating channel transport capacity constraints and the time requirements of different parcels into the model, this research can be more closely related to reality, allowing for the full utilization of high-speed rail transportation capacity without affecting passenger transportation.

## Supporting information

S1 Data (XLSX)
